# Hemoperitoneum as a Life-Threatening Complication of an Acute Cholecystitis in a Patient with Hemophilia A with Inhibitors: A Case Report

**DOI:** 10.3390/healthcare10091652

**Published:** 2022-08-30

**Authors:** Oana Viola Badulescu, Adelina Papancea, Nina Filip, Bogdan Mihnea Ciuntu, Ciprian Cirdeiu, Gabriela Bordeianu, Dan Vintila, Minerva Codruta Badescu, Manuela Ciocoiu, Stefan Octavian Georgescu

**Affiliations:** 1 Department of Pathophysiology, Morpho-Functional Sciences (II), Faculty of Medicine, “Grigore T. Popa” University of Medicine and Pharmacy, 700115 Iasi, Romania; 2 Department of General Surgery, Faculty of Medicine, Grigore T. Popa University of Medicine and Pharmacy, 16 Universitatii Street, 700115 Iasi, Romania; 3 Department of Biochemistry, Morpho-Functional Sciences (II), Faculty of Medicine, “Grigore T. Popa” University of Medicine and Pharmacy, 700020 Iasi, Romania; 4 General Surgery Clinic, “St. Spiridon” County Emergency Clinical Hospital, 1 Independence Boulevard, 700111 Iași, Romania; 5 Department of Internal Medicine, Faculty of Medicine, “Grigore T. Popa” University of Medicine and Pharmacy, 700115 Iasi, Romania

**Keywords:** hemophilia A, bleeding disorders, coagulation, hemostasis

## Abstract

We present the case of a 52-year-old male with severe hemophilia A with inhibitors, who was diagnosticated with acute lithiasic cholecystitis that required surgical intervention due to lack of favorable response to conservatory treatment. During surgery, hemostatic support was performed with activated recombinant factor VII (rFVIIa, NovoSeven^®^). The surgery was performed first laparoscopically with adhesiolysis, followed by subcostal laparotomy and cholecystectomy because of the findings of a pericholecystic plastron with abscess and massive inflammatory anatomical modifications. The patient presented postoperative complications, requiring a second surgical intervention, due to the installation of a hemoperitoneum. Hemostatic treatment with rFVIIa was given for a further 3 weeks postoperatively, and the patient was discharged in safe condition. A surgical intervention increases the risk of bleeding in hemophilic patients, which may have vital complications in the absence of adequate hemostatic support and the support of a multidisciplinary team with experience in hemophilic surgery.

## 1. Introduction

Acute cholecystitis is an acute inflammation of the gallbladder wall, coexisting in most cases with an obstacle to bile flow, causing an acute mechanical-inflammatory pathology. Acute cholecystitis is the most common complication of gallstones and can occur in people with a history of gallstones or can start as the first symptom of gallstones. The clinical symptoms are varied, with patients presenting pain in the right hypochondrium, loss of appetite, nausea, vomiting, febrile syndrome accompanied by chills, headache, or sclero-skin jaundice in severe forms. The diagnosis of cholecystitis is established on the basis of anamnesis, clinical consultation, and paraclinical investigations represented by laboratory investigations and imaging explorations, the most frequently used being abdominal ultrasound and computed tomography.

Hemophilia A is characterized by a lack of coagulation factor VIII, an important factor of the intrinsic pathway of coagulation. The incidence of hemophilia is about 1 in 5000 males; of the affected men, about 80–85% have hemophilia type A, and the rest have hemophilia type B [1]; inhibitors of factor VIII are present in 25–30% of cases with a severe form of the disease [2]. Hemophilia is a coagulopathy that is characterized by prolonged or spontaneous bleeding manifested as hemarthrosis, hematomas, hemoperitoneum, and hematomas of the intestinal wall [3,4,5,6,7]. 

The studies show a mortality of about 3% in the case of acute cholecystitis; this rate of mortality can be increased by patient comorbidity or age [8]. These two associated pathologies represent a major risk of mortality without the availability of coagulation factor and proper multidisciplinary management. 

We highlight the importance of an early diagnosis as well as the management of complications in the case of a patient with a severe form of hemophilia A with inhibitors (anti-factor VIII antibodies = 26 Bethesda units/mL) who sustained a major surgery intervention for acute lithiasic cholecystitis complicated with pericholecystic plastron with abscess and also a reintervention for precocious hemoperitoneum. 

## 2. Case Presentation

A 52-year-old male patient with a history of hemophilia A (factor VIII activity level less than 1%, reference range (RR) = 50–150%) with inhibitors present was admitted to the Hematology Department of the Regional Emergency Hospital “St. Spyridon” Iasi, following three days of increasingly severe upper abdominal pain with a colicative character. The medical history of the patient includes multiple hemophilic arthropathy and chronic hepatitis with type B and C virus, secondary to previous plasma transfusion treatment.

The surgical consult at clinical examination revealed pain on palpation in the upper right quadrant with no evidence of peritoneal irritation or a positive Murphy’s sign.

Laboratory analysis was performed and revealed the following: acute inflammatory syndrome- leukocytosis 14.000/mmc (RR = 4.0–10.0/mmc), C reactive protein = 9.53 mg/dL (RR = 0.00–0.50 mg/dL), Fibrinogen = 598 mg/dl (RR = 200–450 mg/dL), hepatic cytolysis and cholestasis-aspartate aminotransferase = 38 U/L (RR = 5–34 U/L), alanine aminotransferase = 76 U/L (RR = 2–55 U/L), total bilirubin = 1.04 mg/dL (RR = 0.20–1.20 mg/dL), γ-glutamyl transpeptidase = 97 U/L (RR = 12–64 U/L). The coagulation profile showed an activated partial thromboplastin time (aPTT) of 90.6 s (RR = 24–36 s), and the level of anti-factor VIII antibodies was 26 Bethesda units/mL. 

Abdominal ultrasound findings strongly supported an acute cholecystitis diagnosis: the gallbladder was enlarged (hydrops) with a thick wall and contained gall stones and sludge.

Initially, we instituted a conservative treatment with intravenous antibiotics, antispastic medication, and hemostatic support with rFVIIa (NovoSeven^®^) according to the hematological protocol (90 µg/kgbw) [9]. 

After four days of treatment, the patient was admitted in the surgery clinic, and a surgical intervention by laparoscopic cholecystectomy was planned. Conversion open cholecystectomy was also considered and prepared for in considering the hemorrhagic risk. 

**Figure 1:**
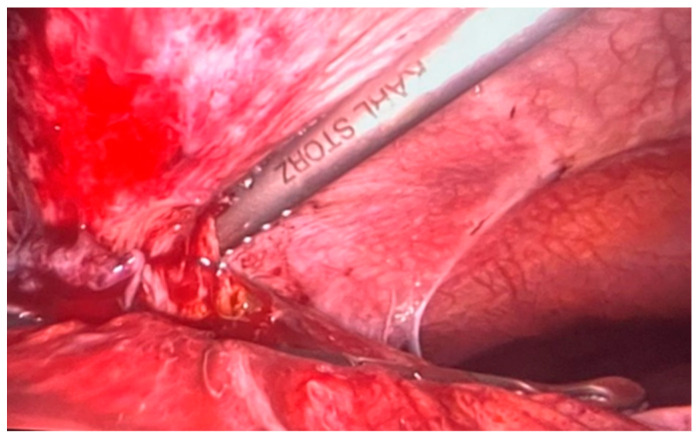
Dissection of the pericholecystic plastron.

The surgery was started immediately after the administration in bolus of the dose of NovoSeven (90 µg/kgbw) by exploratory laparoscopy. The exploration of the abdominal cavity revealed a pericholecystic plastron with the adhesion of the transvers segment of the colon. After a carefully executed dissection, a purulent secretion breached out and was aspirated (Figure 1). 

We proceeded with the dissection, but the inflammation of the tissue was severe, and it modified the anatomy of the subhepatic region. We decided on a conversion to right subcostal incision. The cholecystectomy was performed retrograde after the dissection of the adhesions, with the identification of the cystic channel and the artery that were ligated and sectioned. 

A carefully executed hemostasis was done in the subhepatic region; a hemostatic sponge was placed in the vesicular dissection from the liver, and drainage tubes were placed in the subhepatic region and in the Douglas space. The intervention lasted 3 h and the loss of blood was about 150 mL.

Postoperatively, NovoSeven^®^ was administered every 2 h, at a dose of 90 µg/kgbw, according to the therapeutic protocol [9]. The factor replacement level was maintained between 80% and 100%.

Acute-on-chronic calculous cholecystitis was confirmed by anatomopathological analysis.

**Figure 2:**
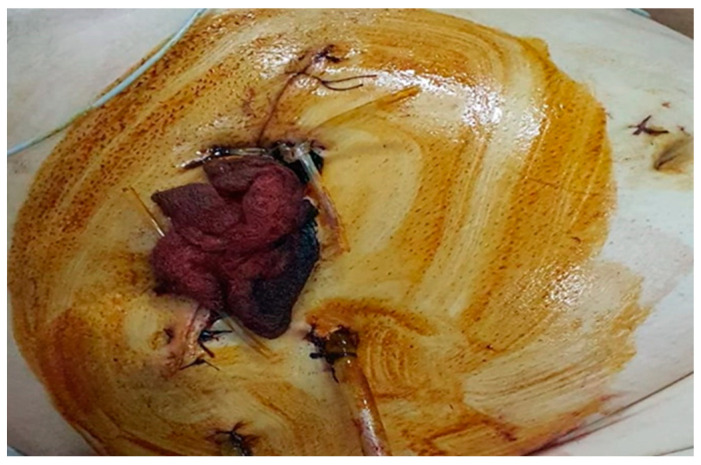
The external extensions of the perihepatic meshes.

Postoperatively, the patient was extubated and admitted to the intensive care unit for monitoring; the NovoSeven^®^ administration continued with the same doses at every 2 h. After 10 h, the patient developed the clinical picture of hypovolemia (pallor, hypotension, tachycardia); palpation revealed increased abdominal tenderness, the drainage was about 500 mL of hematic fluid, and the hemoglobin level decreased from 14 g/dL to 6 g/dL; the abdominal ultrasound was performed in emergency and confirmed the presence of intraperitoneal fluid. We decided upon emergency surgical reintervention due to hemodynamic instability; 500 mL of intraperitoneal blood was evacuated, and a diffuse hemorrhage from the subhepatic region and from the dissection of the inflammatory process was also observed, with no major source of active bleeding. A perihepatic packing was performed (Figure 2).

After the reintervention surgery, the administration of NovoSeven^®^ continued at every 2–4 h in the same doses (90 µg/kgbw) until the seventh postoperative day, with the maintenance of the coagulation factor level between 80–100%.

The hemoglobin value was stationary at 7.4–8.6 mg/dL. The evolution of the patient was favorable, with the removal of the hepatic packing in the seventh day after the reintervention. On the postoperative day, the patient had mild hematic drainage, less than 50 mL that decreased in the following days; the hemoglobin value was stable, and an ultrasound revealed only 20 mL of liquid in the Morison space.

Colistin and Linezolid were prescribed throughout the hospitalization for wound infection with *Acinetobacter baumannii* and *Enterococcus faecium*.

The favorable evolution of the patient, from the point of view of the bleeding phenomena, allowed the administration of the hemostatic treatment with NovoSeven^®^ from the eighth postoperative day, at every 6–12 h, in doses of 90 µg/kgbw until discharge (day 23 postoperatively). On the 19th postoperative day, the drain was removed.

**Figure 3:**
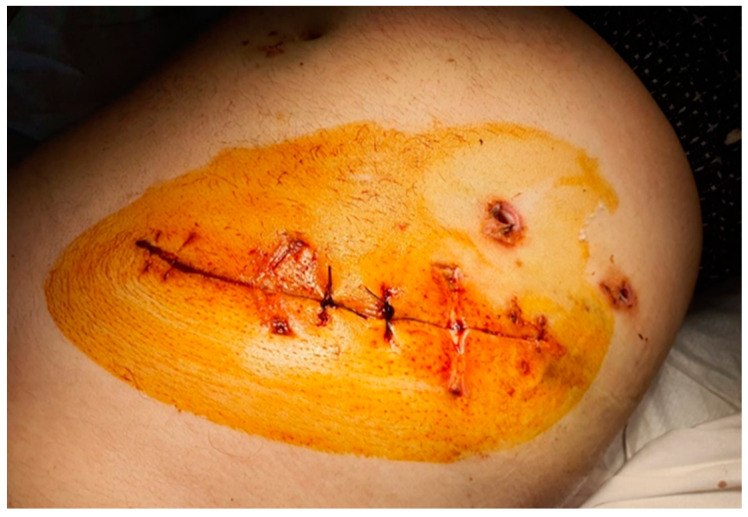
Incisional site after complete healing (15th day post intervention).

The patient recovered and was discharged in safe condition, without hemorrhagic phenomena, with a hemoglobin value of 11 g/dL. He received home treatment with Novoseven^®^ for the prophylaxis of hemorrhagic phenomena (Figure 3).

## 3. Discussion

The patient’s standard therapy does not work in cases where there is inhibitors acquirement and the risk of bleeding is high. Coagulation factor replacement therapies supplement FVIIIa deficiency, but approximately 30% of treated patients develop inhibitors [3,10]. There are a number of factors that influence the appearance of inhibitors, such as ethnic background, genetic mutations, and previous exposure days to treatment. The risk of inhibitors increases with age [11].

The development of inhibitors in hemophilic patients complicates the bleeding events management, as standard replacement approaches are no longer effective [12]. In the presence of FVIII inhibitors, the binding of activated FVIII of coagulation to activated FIX of coagulation or FX of coagulation is no longer possible, and FX thus remains inactive [13]. This situation requires the administration of by-pass agents (BPAs), which act on the extrinsic coagulation pathway, bypassing the intrinsic pathway. Leissinger et al. [12] mentioned that prophylaxis with a bypass agent reduces joint bleeding and other types of bleeding compared to on-demand bypass therapy.

However, despite treatment with hemostatic BPA, administered on demand or for prophylactic purposes, very few hemophilic patients with inhibitors have zero episodes of bleeding [14]. This was concluded in the prospective, multi-centric, non-interventional study of Mahlangu J. and al. [14]. The findings of this study demonstrate the need for more effective treatments to prevent or reduce bleeding in this patient group. In the same study, it is mentioned that increasing patients’ adherence to prophylactic treatment through an innovative therapy that reduces the inconveniences of current therapy (difficulty approaching the venous bed, frequent administration that takes a long time) would play a key role in preventing bleeding.

Non-factor therapies developed in recent years would be the best solution to prevent bleeding in patients with inhibitors. It seems that therapy with Emicizumab, a bispecific monoclonal antibody, would eliminate the inconvenience of administering bypass agents. Uncommon subcutaneous administration (CPR) (once a week, once every 2 or 4 weeks) and the superior hemostatic effect compared to bypass agents is currently the gold standard in the prophylaxis of bleeding in patients with inhibitors [15]. The high costs, however, represent the main obstacle for countries with a medium level of development in accessing this type of treatment.

Our patient, known to have a severe form of hemophilia with inhibitors, and a high-responder, was receiving on-demand treatment with activated recombinant alpha eptacog (NovoSeven) for the treatment of bleeding episodes, and he had not been hospitalized for bleedings in the last three years.

The patient developed an acute lithiasic cholecystitis with pericholecystic plastron with abscess that needed classic surgery and a reintervention for bleeding, thus presenting vital postoperative complications. The extension of the pericholecystic inflammatory process and the severe coagulopathy led to a medium diffuse intra-abdominal hemorrhage that needed hemostasis through perihepatic packing. Adequate replacement therapy with coagulation factor from hospitalization, intraoperative and post-intervention, was crucial for the good evolution of the patient due to the possibility of access to the required amount. The patient was hospitalized for 33 days. During this period, he received 840 vials of NovoSeven^®^, and 8 units of erythrocyte mass were required for transfusion treatment.

A similar case of cholecystectomy in a young hemophilic patient with inhibitors was reported in 2021 by Giacometto and al. [16]. Unlike our patient, he was receiving prophylactic treatment with Emicizumab. During the surgery, treatment with rFVIIa was added, and after one week post operation, the patient was discharged without presenting hemorrhagic phenomena. The lack of postoperative complications and the very good evolution of patient was probably influenced by prophylactic treatment with Emicizumab [16].

The hemostatic effects of prophylactic treatment with Emicizumab were highlighted by Oldenburg J and al. [15] in the phase 3, multicenter trial Haven 1. The main objective of this study was to compare the bleeding rate between patients who took prophylactic treatment with Emicizumab and those who did not receive prophylaxis. Emicizumab treatment was given subcutaneously once a week. The annual bleeding rate showed less events in patients receiving prophylactic treatment with Emicizumab compared with 23.3 events in those without prophylaxis, representing a difference of 87% in favor of Emicizumab prophylaxis. Moreover, 22 participants in the Emicizumab prophylaxis group (63%) had zero bleeding events, compared with 1 participant (6%) in the no prophylaxis group. Regarding bypass agent prophylaxis in hemophilic patients with inhibitors, studies have shown that prophylaxis with rFVIIa or aPCC reduced the number of episodes of hemarthrosis and other bleeding and improved the quality 58–60 patients’ lives compared to on-demand bypass therapy [17,18,19].

In the study of prophylaxis with rFVIIa, the frequency of bleeding was reduced by 45% with a dose of 90 µg/kg administered daily compared to 59% for the dose of 270 µg/kg, an insignificant difference [17].

The results of the published studies raise the hypothesis that the life-threatening complications developed postoperatively in our patient, due to the lack of prophylactic substitution treatment as well as low patient compliance with periodic evaluation of the disease.

Poor adherence to treatment is a major impediment in the administration of the correct prophylactic treatment to patients with hemophilia type A and B without inhibitors, and implicitly in the prevention of bleeding episodes.

A global study of practice patterns identified the following as causes of poor adherence: denial of the disease, lack of family commitment, lack of understanding of the beneficial effects of prophylactic treatment, and lack of time to go to the hospital and allocate infusions [20].

Although similar surveys have not been performed in hemophilic patients with inhibitors requiring prophylactic treatment with BPA, the same problems with a negative impact on patient adherence to treatment are certainly found. Continuing education and the support of the medical team are essential to encourage patients and families to be aware of the importance of prophylactic treatment in preventing and reducing the number of bleeding episodes [12].

The lack of adherence to the treatment of our patient, who had not been present for three years for hematological evaluation, was a factor favoring postoperative complications.

The costs of such an emergency surgery are very expansive, primarily due to the costs of the NovoSeven^®^ coagulation factor treatment to which is added the expenses related to hospitalization, sanitary materials, laboratory tests, and other drugs utilized during the hospitalization.

The cost of prophylactic treatment with coagulation factors is extremely expensive, as mentioned by Leissinger et al. [12].

FVIII prophylaxis for patient without inhibitors was reported as being 2.4–3.1 higher than on-demand therapy [21]. It is worth mentioning that the aforementioned prices are brute figures that ignore potential financial savings such as fewer hospitalization days, medical leaves, or complication-related costs.

The cost of daily prophylaxis with rFVIIa has not been reported, but it is certainly higher.

However, the costs should be interpreted in comparison with the costs of a single episode of major bleeding with lethal potential, as is the case with our patient, with the benefits of reducing the number of days of hospitalization, days of absence from work, and treatment costs potentially disabling complications such as arthropathy requiring total stents.

They also do not take into account the improved quality of life of the hemophiliac patient reported by patients receiving prophylaxis [17,22,23].

The total cost of the surgery for our patients exceeded 500,000 euros. The high cost of surgery for hemophilic patients was also mentioned by Jason D. Wals and colleagues [24]. They reported two cholecystectomies in two patients with acquired hemophilia and anti-factor VIII antibodies present; the cost of each surgery exceeded $50,000, but no patient had complications or hemorrhagic events, which would impose an increased factor consumption and imply additional costs [24].

Despite the very high costs, we were able to successfully treat our patients with rFVIIa and obtain dramatic hemostatic results using the standard dosage (90 μg/kgbw) and timing until the bleeding was stopped. As we were surrounded by lots of limitations, this can be considered a great achievement for our hospital. Despite prophylactic replacement therapy with a role in the prophylaxis and reduction of bleeding episodes, the by-pass agents, such as rFVIIa, represent the main therapy in the surgery management of the hemostasis of hemophilic patients with inhibitors.

rFVIIa intervenes in hemostasis. Increased thrombin production increases platelet aggregation, which leads to the activation of FXIII coagulation and ensures the production of a stable fibrin clot. The half-life of rFVII is 2–3 h.

## 4. Conclusions

Access to hemostatic support with NovoSeven^®^ through the National Health Program for Hemophilic Patients was the main element supporting the possibility of performing surgery in these patients and ultimately saving the life of the patient presented in this report. Surgery in hemophilic patients should be performed in specialized centers that have access to national health programs for treatment with coagulation factor and that have doctors with experience in hemophilic surgery. Interdisciplinary management by surgeons, hematologists, and anesthesiologists and appropriate hematologic intervention are mandatory to achieve a successful outcome in surgery of hemophilic patients.

## Data Availability

All data generated or analyzed are included in this case report. Further enquiries can be directed to the corresponding author.
